# Mesenchymal stem cells and acellular products attenuate murine induced colitis

**DOI:** 10.1186/s13287-020-02025-7

**Published:** 2020-11-30

**Authors:** Yan Li, Jessica Altemus, Amy L. Lightner

**Affiliations:** 1 Department of Colorectal Surgery, Digestive Disease Surgical Institute, Cleveland Clinic, 9500 Euclid Ave, Cleveland, OH 44195 USA; 2 Department of Inflammation and Immunity, Lerner Research Institute, Cleveland Clinic, Cleveland, OH USA

**Keywords:** Mesenchymal stem cells, Extracellular vesicles, Inflammatory bowel disease, Murine colitis model, Therapy

## Abstract

**Background:**

Mesenchymal stem cells (MSCs) are a well-established immunomodulatory agent which can also promote tissue repair and regeneration. Recent studies have demonstrated MSCs as a novel therapeutic for inflammatory bowel disease (IBD), a chronic idiopathic inflammatory disorder of the gastrointestinal tract. However, the precise role of MSCs in regulating immune responses is controversial, and its significance in the pathogenesis remains IBD undefined. In addition, MSCs’ acellular product, extracellular vesicles (EVs), may also play an important role in the armamentarium of therapeutics, but how EVs compare to MSCs remains unknown due to the lack of side-by-side comparative investigation. We herein compared MSCs and MSC-derived EVs for the treatment of IBD using a DSS-induced colitis model.

**Methods:**

A DSS-induced colitis model was used. At day 4, mice received adipose-derived MSCs, MSC-derived EVs, or placebo. Weight loss, stool consistency, and hematochezia was charted. At day 8, murine colons were harvested, histologic analysis performed, and serum/tissue cytokine analysis conducted.

**Results:**

MSCs and EVs demonstrated equivalent immunosuppressive function in DSS-treated mice through decreased colonic lymphocyte infiltration and attenuated disease severity after both MSC and EV treatment. Furthermore, both MSCs and EVs have an equivalent ability to inhibit inflammation in the DSS colitis model by inhibiting JAK, JNK 1/2, and STAT3 signaling.

**Conclusions:**

These results suggest that (i) both MSCs and EVs are effective therapeutic candidates for a DSS-induced mouse colitis model, (ii) MSCs and EVs have similar immunosuppressive and anti-inflammatory functions, and (iii) EVs may present a novel future therapeutic for the treatment of IBD.

## Introduction

Ulcerative colitis (UC) and Crohn’s disease (CD) are the two primary phenotypes of inflammatory bowel disease (IBD), a chronic idiopathic inflammatory disorder of the gastrointestinal tract [1–4]. Patients suffer from a remitting-relapsing disease course and are often initiated on immunomodulator and/or monoclonal antibodies to address their symptoms. However, monoclonal antibodies are limited by lack of primary response [5–9], loss of secondary response [10, 11], and increased risk of serious opportunistic infections [12]. In addition, despite the advent of monoclonal antibodies with infliximab approval in 1998, up to 30% of UC patients and 80% of CD patients still require surgical resection to alleviate symptoms related to progressive bowel wall damage [2, 4]. Despite the increasing incidence of IBD worldwide, and the already affected 3.1 million people in the USA, there is still no medical or surgical cure for IBD; additionally, the pathophysiology remains largely unknown. Therefore, there is an unwavering desire to better understand the pathophysiology of IBD in order to design better therapeutics addressing the underlying pathophysiology.

A growing number of clinical trials are utilizing healthy adult donor mesenchymal stem cells (MSCs) to treat CD [13–17]. MSCs are thought to act as an anti-inflammatory and immunosuppressive therapy which can migrate to sites of inflammation and injury, and promote tissue repair [18–22]. These cells have proven to be safe and effective in the local treatment of Crohn’s perianal fistulizing disease [13–17], and early data from limited studies suggest MSCs may also be effective for the treatment of intestinal UC and CD [22, 23]. However, there are still several limitations to cell-based therapies including scalability of manufacturing, infrastructure for cell manufacturing, optimal modes of cell delivery, and significant cost burdens.

MSC-derived extracellular vesicles (EVs) may provide a solution to the aforementioned limitations of cell-based therapy. MSCs are thought to exert their beneficial effects in tissue generation in a paracrine fashion rather than actual engraftment into tissue [23–26]. Most of the paracrine function of MSCs is attributed to the secretion of EVs, membrane-bound particles, carrying protein, mRNA, and miRNA. EVs, hosting the functional aspects of MSCs, can infiltrate local tissues or travel systemically to sites of inflammation while signaling to other cells [27–30]. EVs have already been shown to reverse acute kidney injury, vascular injury, pulmonary hypertension, and obesity [16, 25, 31–33], but colitis has only recently been evaluated. If EVs were equally effective as MSCs for colitis, one could better understand the mechanism of MSC healing and begin to explore acellular therapies for IBD. We therefore sought to better investigate the role of EVs as a therapeutic alternative to MSCs in IBD by comparing MSCs to MSC-derived EVs in a murine model of colitis. The two primary outcomes of interest were to (1) understand MSC-derived EVs’ ability to reverse colitis in comparison to MSCs and (2) propose MSC-derived EVs’ mechanism of action in treating colitis.

## Materials and methods

### Ethics permission

This study was approved by the Cleveland Clinic’s Institutional Review Board (IRB) (Ethical Approval 19-908).

### Animals

Wild-type (WT) mice (C57Bl6 background) were maintained under pathogen-free conditions in the animal facility of Lerner Research Institute, Cleveland Clinic, Cleveland, OH. All animal handling and associated procedures were approved by the Institutional Animal Care and Use Committee of Cleveland Clinic, and all were done in accordance with the United States Department of Health and Human Services Guide for the Care and Use of Laboratory Animals and institutional guidelines.

### Induction and assessment of disease severity of colitis

Dextran sulfate sodium (DSS)-induced colitis has been widely used as an experimental model to study pathogenic mechanisms underlying IBD, and disease severity was assessed by assigning clinical scores by following previously published protocols [34–36]. In brief, on day 0, 3% DSS (MW 40 kDa; Sigma-Aldrich, St. Louis, MO) was added to the drinking water. Mice were weighed daily and inspected visually for any sign of sickness. The presence of blood in the stool was tested by Hemoccult II Sensa Fecal Occult Blood Test (Beckman Coulter, Brea, CA) every other day. Previously published literature described that the mice would develop disease on days 3 to 5 (clinical score = 1, scoring charts listed below) [34, 36]. The delivery of MSCs, EVs, or normal saline (PBS control) was performed on day 4. DSS was continued. Four days after MSC, EV, or placebo injection (8 days from initiation of DSS), the mice were sacrificed by carbon dioxide followed by cervical dislocation, and their colons were collected for histological analysis. Differences in serum and intestinal levels of inflammatory factors between PBS control and MSC- or EV-treated mice were compared. The serum collected from the mice after the sacrifice was diluted and the measurement levels of total systemic cytokines calculated by using commercial kits (BioLegend, San Diego, CA) following manufacturer-provided protocols.

### Evaluation of colitis severity

To grade the clinical severity of DSS-induced colitis, mice were assessed for body weight (daily), stool consistency (daily), and hematochezia by fecal occult blood test (every other day) with clinical scores as described in Table 1.

**Table 1:** Body weight (daily), stool consistency (daily), and hematochezia of mice by fecal occult blood test (every other day) with clinical scores

Clinical score	Weight loss (%)	Stool consistency	Hematochezia
0	None	Normal	None
1	1–10%	Soft stool	Hemaoccult positive
2	10–20%	Diarrhea	Gross blood
3	20%	Diarrhea	n/a

### Isolation of adipose-derived mesenchymal stem cells

Following institutional review board approval (IRB) and written informed patient consent for tissue collection as part of the regenerative medicine patient identified biobank, abdominal subcutaneous *adipose-derived MSCs* were isolated following the protocol described in the previous study with minor modifications [37–39]. Briefly, healthy human adult subcutaneous adipose tissue was obtained during open ventral hernia repairs of healthy patients defined as no history of malignancy or IBD. The subcutaneous adipose pearls were micro-dissected, washed in phosphate-buffered saline (PBS) to remove erythrocytes, minced, and digested with 0.25% type I collagenase (Gibco Life Technologies, Waltman, MA) at 37 °C for 60 min under constant shaking. Cells were pelleted, supernatant was removed, and cells were resuspended and cultured in xeno-free, serum-free MSC NutriStem® XF Medium (Biological Industries USA, Cromwell, CT, cat# 05-200-1A-KT) under standard cell culture conditions of 37 °C/5% CO_2_. The cultured MSCs were grown to 85–90% confluency in T75 tissue culture flasks before passaging. MSCs were finally harvested at passage 3 to 4.

To demonstrate the colonic distribution of the injected MSCs, MSCs labeled with CFSE Cell-Labeling Solution (Life Technologies, Carlsbad, CA) were injected into the peritoneal cavity of the mouse. After sacrifice, the colon tissues were harvested to make cryosections for examination under a fluorescence microscope (Leica Microsystems, Buffalo Grove, IL).

### Extracellular vesicle isolation and identification

To prepare EVs, the previously listed medium(s) from MSC culture was ultra-centrifuged for 16 h at 100,000×*g* at 4 °C in a 45Ti fixed angle rotor using polycarbonate tubes (Beckman Coulter, Brea, CA). After ultracentrifugation, the top layer medium suspension was harvested, filtered with a 0.22-μm PES filter, and stored at 4 °C. The MSC-derived EVs were extracted and concentrated from MSC cultured media. Briefly, during the MSC harvest procedure, the cultured media were collected and filtered through a 0.22-μm filter to remove cell debris and large vesicles, followed by ultracentrifugation at 30,000×*g* for 20 min to pellet larger microvesicles. The supernatants were then subjected to ultracentrifugation at 120,000×g for 3 h to sediment the EVs. The resulting pellets were resuspended in PBS for injection purposes. The identification of the EVs was determined by Zetaview Nanoparticle Tracking Analyzer (Munich, Germany) and its corresponding software (ZetaView 8), by following the established protocol [40]. In brief, each sample was mixed with 1x filtered PBS in 1:2 dilution, and the instrument measured each loading at 11 different positions, with two cycles of readings at each position. Three different buffer/media (PBS control, fresh media, and MSC media) were used for EVs comparatively analyzed by using ZetaView (Fig. S1). The isolated MSC particles’ average diameters are 100 nm, were within the expected size range for EVs (90–120 nm), and significantly greater diameter than PBS (~ 43 nm) and media control group (~ 90 nm). To demonstrate the distribution of the injected EVs in the colon, EVs labeled with CFSE Cell-Labeling Solution (Life Technologies, Carlsbad, CA) were injected into the peritoneal cavity of the mouse. To fluorescently label EV proteins, the isolated EVs were incubated in 100 nM to 10 μM CFSE (BioLegend, San Diego, CA) for 30 to 45 min at 37 °C in the dark. After sacrifice, the colon tissues were harvested to make cryosections for examination under a fluorescence microscope (Leica Microsystems, Buffalo Grove, IL).

### Histopathological analysis

Proximal and distal colonic sections were fixed in 4% paraformaldehyde and embedded in optimal cutting temperature (O.C.T.). The 5-mm sections were stained with hematoxylin and eosin (H&E). The microscopic colonic epithelial damage and inflammation severity [41, 42] were assigned scores as follows: 0 = normal; 1 = hyperproliferation, irregular crypts, and goblet cell loss; 2 = mild to moderate crypt loss (10%–50%); and 3 = severe crypt loss.

### Systemic and local cytokine production

Blood was collected from the mice of MSC, EV, and control groups at the end of the experiment and centrifuged for 10 min at 800×*g* at 4 °C. The sera were then frozen and stored at − 80 °C until further examination. The colon tissue was mechanically homogenized in RIPA lysis buffer (Santa Cruz Biotechnology, Dallas, TX) containing a mixture of protease inhibitors (Santa Cruz Biotechnology, Dallas, TX). The homogenized tissue was incubated on ice for 30 min, with brief vortexing every 5 min. Tissue lysates were centrifuged at 13,000 rpm for 30 min at 4 °C, the pellets were discarded, and protein concentration of the supernatant was measured using the Pierce BCA Protein Assay kit (Waltman, MA). For the detection of cytokine levels, 96-well high binding plates were coated with 2 μg/ml anti-mouse IL-6, IL-10, TNF-α, IFN-γ, IL-17, or IL-12 (BioLegend, San Diego, CA) diluted in PBS. Protein lysates (diluted 1:20) or serum (diluted 1:50) were loaded onto the coated ELISA plate. Bound cytokines were detected using biotin anti-mouse IL-6, IL-10, TNF-α, IFN-γ, IL-17, or IL-12 (BioLegend, San Diego, CA) and subsequent avidin-HRP antibodies (BioLegend, San Diego, CA). The ELISA color reaction was initiated using tetramethylbenzidine (TMB) substrate (Thermo Scientific, Waltman, MA). 2 M H_2_SO_4_ was used to stop the TMB reaction, and absorbance at 450 nm was measured. The colon local cytokine concentrations were normalized to the starting initial protein concentration.

### Western blotting and signaling determination

The protein lysates from colonic tissue were mixed with 2x Laemmli buffer and separated according to their molecular mass on 4–20% SDS-PAGE gel and transferred to a nitrocellulose membrane (Bio-Rad, Hercules, CA). Western blot and antibody detection were performed by established protocol. Rabbit anti-STAT3 (4904), anti-JNK (9252), anti-JAK1 (3344), and anti-JAK2 (3230) antibodies were purchased from Cell Signaling Technologies (Danvers, MA) and mouse Direct HRP anti-β-actin (664803) was purchased from BioLegend (San Diego, CA).

### Hyperbaric oxygen-related tissue damage analysis

Superoxide dismutase (SOD) were employed for the hyperbaric oxygen-related tissue damage analysis. Lipid peroxidation levels were measured by SOD Assay Kit (Cayman Chemicals, Ann Arbor, MI) according to the manufacturer’s instructions. Absorption was recorded at 450 nm. The SOD levels were expressed as nmol/mg protein.

### Statistical analysis

All listed experiments were repeated at least twice with similar results. To determine whether statistically significant differences existed between groups, data were analyzed by nonparametric Kruskal-Wallis ANOVA. Further analysis by using two-way ANOVA test or, if group variances were dissimilar, Bonferroni-corrected multiple *t* tests, produced outcomes similar to those of Kruskal-Wallis ANOVA. A *p* value of < 0.05 was considered significant.

## Results

### MSCs and EVs reverse colitis progression in the DSS-induced colitis mouse model

**Fig. 1:**
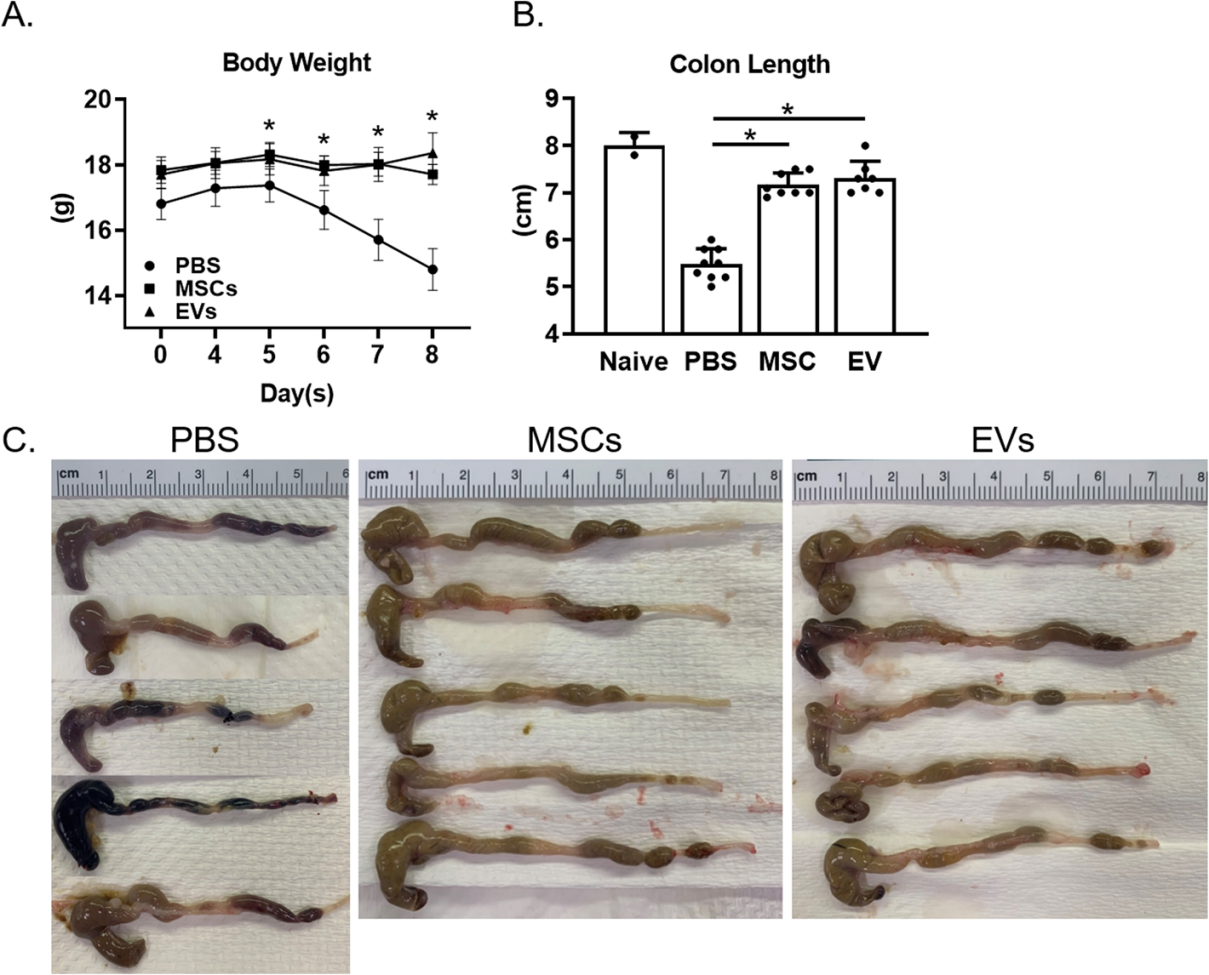
MSCs and EVs protected mice from DSS-induced colitis. **a** Clinical body weight scores of the PBS/MSC/EV-treated mice. Combined results from two experiments. *n* = 9. *p < 0.05. **b** The colon length of DSS-induced colitis groups treated by PBS/MSCs/EVs after sacrifice. The colon length of all PBS mice was significantly shorter than that of MSC/EV-treated groups. **c** The pictures of mouse colons, dissected from the cecum to the anus. Data are represented as mean ± SEM, *p < 0.05

Colitis was induced in wild-type mice (C57Bl6 background) by adding 3% DSS to the drinking water. The development of colitis was assessed daily by monitoring clinical scores. Once mice showed mild clinical signs of colitis (day 4), we treated the mice with 10 million human adipose-derived MSCs, EVs (supernatant from 10 million human adipose-derived MSCs), or PBS (control) in 500 μl volume by i.p. injection. The mice were then monitored continuously for 4 days. The disease activity index score on day 8 was significantly reduced in the MSC- and EV-treated mice compared to control PBS mice (Table 2). We carried out immunological and histopathological assays as described. MSCs and EVs showed equivalent efficacy in treating DSS developed severe colitis (Fig. 1a). Compared with the severe colitis in the PBS-treated mice, progression of colitis was stopped in both MSC- and EV-treated mice, demonstrated by the longer colonic lengths 4 days after treatment; the mean colon length of the PBS control group (5.48 ± 0.33 cm) was significantly shorter than that of MSC-treated (7.18 ± 0.25 cm) and EV-treated (7.31 ± 0.35 cm) groups (Fig. 1b, c; *p* < 0.05).

**Table 2:** Disease activity index score

Group	D0	D4	D5	D6	D7	D8
PBS	0	0.55 ± 0.21	0.94 ± 0.32	2.25 ± 0.29	2.74 ± 0.31	2.92 ± 0.15
MSCs	0	0.25 ± 0.11^a^	0.57 ± 0.28^a^	0.66 ± 0.2^a^	0.79 ± 0.11^a^	1.25 ± 0.51^a^
EVs	0	0.31 ± 0.05^a^	0.62 ± 0.15^a^	0.74 ± 0.19^a^	1.01 ± 0.17^a^	1.24 ± 0.44^a^

Values are presented as mean only or mean ± SEM. *D*, day of dextran sodium sulfate (DSS) administration. ^a^*p* < 0.05

### Tracking peritoneal cavity delivered MSCs or EVs in the mouse

**Fig. 2:**
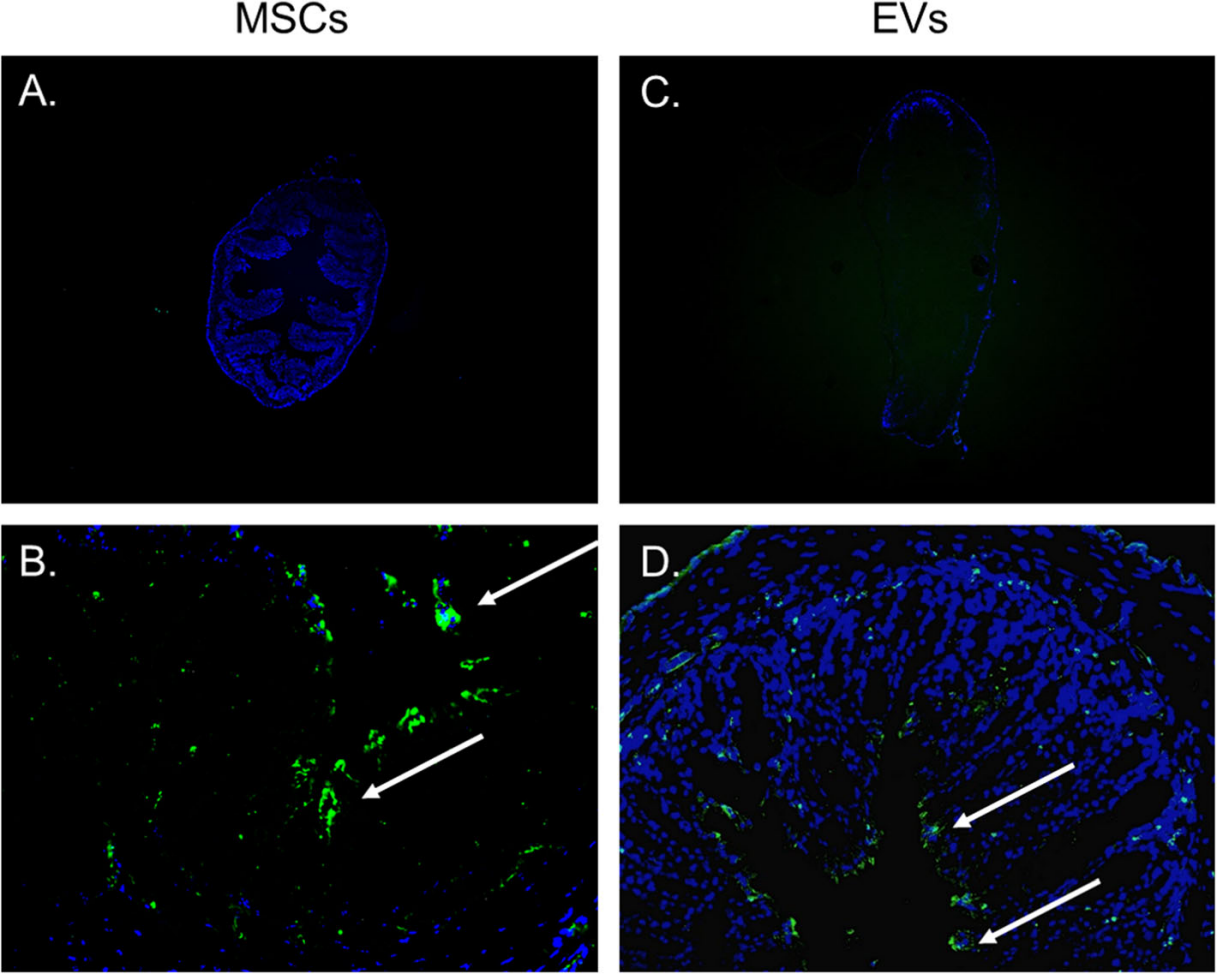
Local delivery of MSCs/EVs into mouse by i.p. injection. **a**, **b** MSCs. **c**, **d** EVs. For each mouse, CFSE-labeled MSCs or EVs were injected into the peritoneal cavity. At sacrifice, colon cryosections were prepared to examine the distribution of the injected MSCs/EVs (green) under a fluorescence microscope at original magnifications × 40 (**a**, **c**) and × 200 (**b**, **d**) with DAPI (blue). Arrows highlighted the CFSE-labeled MSCs/EVs

To determine whether MSCs or EVs directly suppress the local inflammatory responses in vivo, we sacrificed two pairs of mice with developed colitis at 24 h post-MSC or EV injection. In brief, we randomly selected two pairs of DSS-induced colitis mice treated by fluorescently MSCs or EVs, to demonstrate whether the MSCs or EVs had migrated to the area of inflammation. At sacrifice, we removed the colon for cryosections and examined them for the presence of MSCs or EVs under a fluorescence microscope. MSCs/EVs (green) were present at 24 h after injection. (Fig. 2). This data shows that MSCs and EVs can migrate to and throughout the colon without the need for subsequent injections in mice.

### MSCs and EVs inhibit local inflammation

**Fig. 3:**
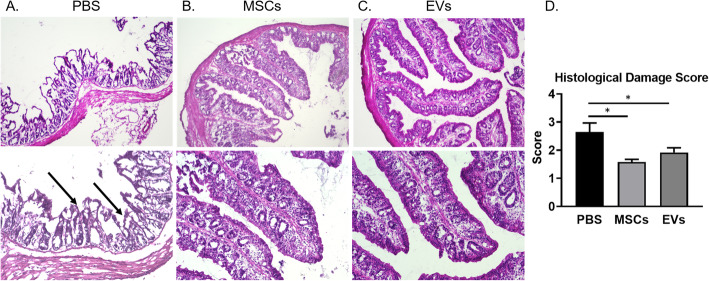
Representative histopathologic image findings of the colonic mucosa (H&E). Mucosa of the PBS control group (**a**), MSCs (**b**), and EVs (**c**); arrows mark the infiltration of inflammatory cells and nearly total loss of crypts at colonic mucosa of the PBS group. Mice had markedly reduced leukocyte infiltration in MSC and EV groups as assessed by H&E staining (magnification: upper, × 100; arrows shown at lower, × 200). **d** Histological damage score was calculated based on microscopic colonic epithelial damage and infiltration of inflammatory cells, and the microscopic damage score of the PBS group was significantly higher than that of MSC- and EV-treated groups. Data are represented as mean ± SEM, *p < 0.05

Histopathological assays showed markedly decreased colon inflammation (Fig. 3a–c) in the MSC- and EV-treated mice. Decreased damage was observed histologically in the colon mucosal layer of the MSC- and EV-treated groups as compared to the PBS group. The crypts were straight, and the base of the tubular glands reached the muscularis mucosa. The epithelial cell layer on the surface of the mucosa was intact. In contrast, the PBS group showed nearly complete destruction of crypts and infiltration of inflammatory cells. However, ulceration of colonic mucosa was rarely observed in the colonic mucosa of the PBS group.

When the microscopic histological damage score was calculated based on microscopic colonic epithelial damage and infiltration of inflammatory cells, the microscopic damage score of the PBS group was significantly higher than that of MSC- and EV-treated groups (Fig. 3d).

### MSC and EV treatment decreased systemic and local inflammatory cytokines and increased anti-inflammatory cytokine production

**Fig. 4:**
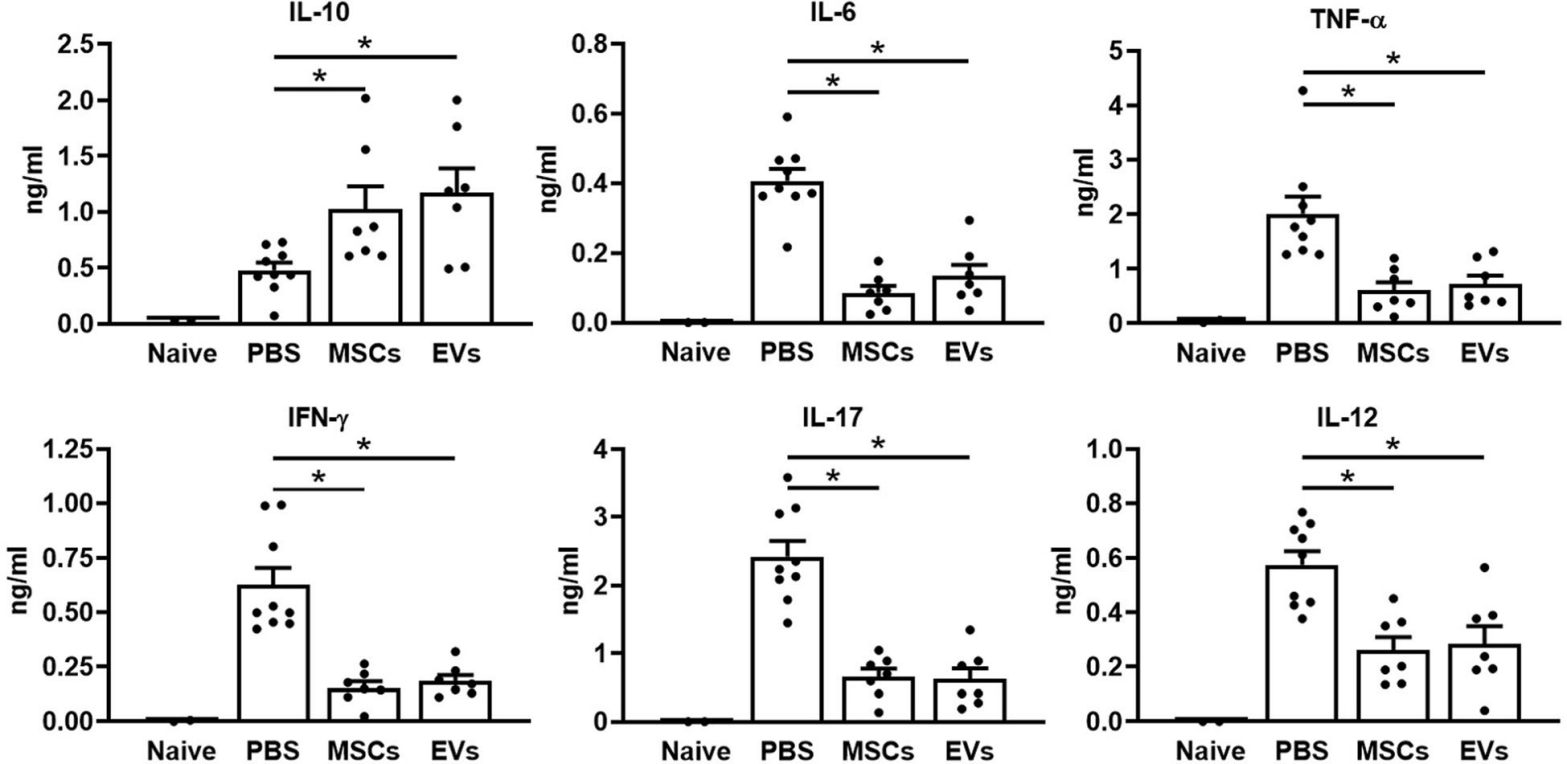
Systemic cytokine production. Mouse serum cytokines were determined by ELISA, and the concentration of IL-6, IL-10, TNF-α, IFN-γ, IL-17, and IL-12 were shown as ng/ml. *n* = 9 in the PBS group, *n* = 7 in MSC/EV groups. Data are represented as mean ± SEM, *p < 0.05

**Fig. 5:**
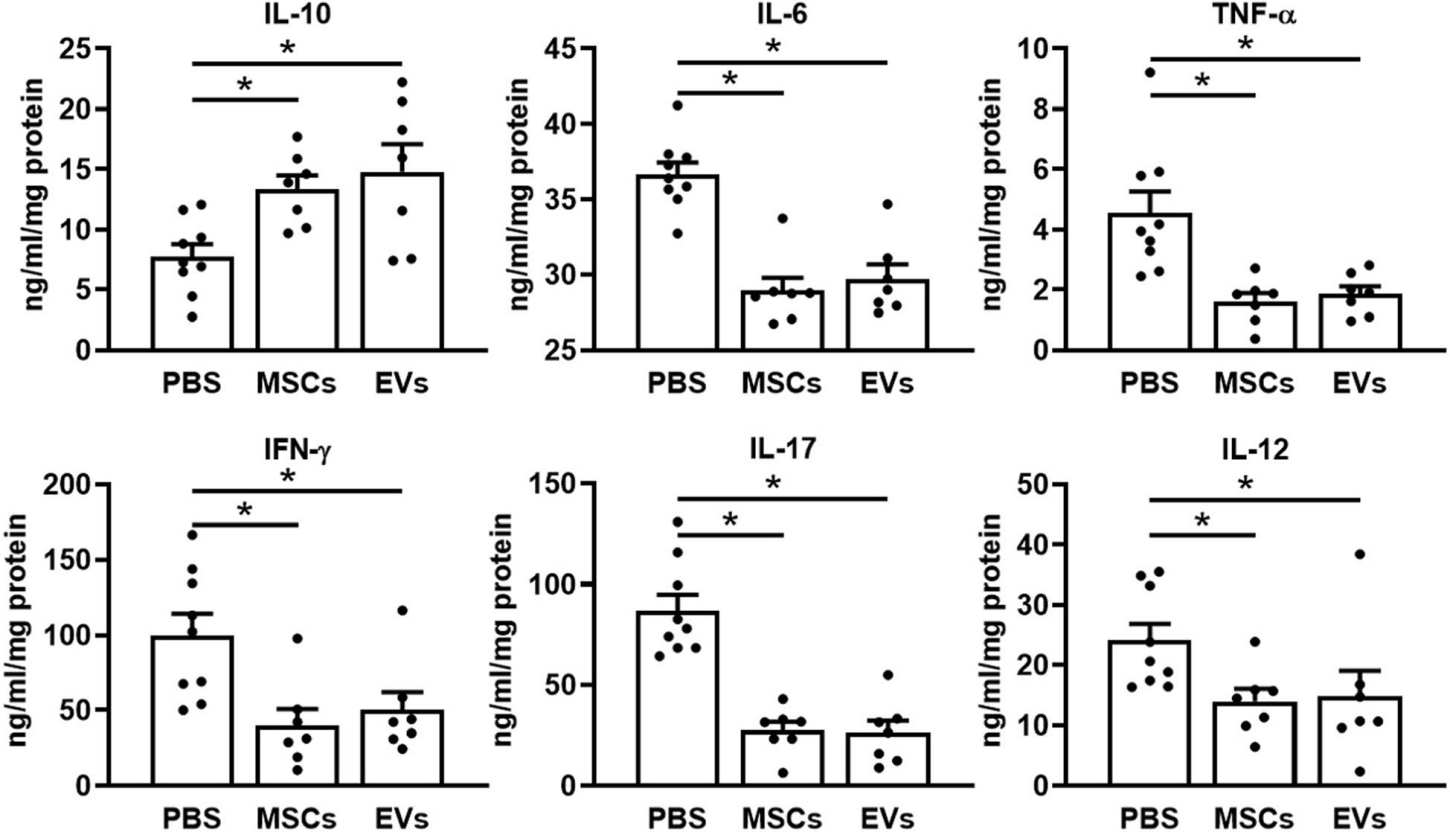
Local cytokine production. Mouse local produced cytokines were tested from protein lysate by ELISA. The colon local cytokines’ (IL-6, IL-10, TNF-α, IFN-γ, IL-17, and IL-12) concentrations were normalized to the starting initial protein concentration, represented as ng/ml/mg protein. *n* = 9 in the PBS group, *n* = 7 in MSC/EV groups. Data are represented as mean ± SEM, *p < 0.05

MSC and EV treatment protects against DSS-induced mouse colitis via the reduction of local and systemic inflammatory responses, thereby leading to limited tissue damage and reduced disease severity. Upon treatment with MSCs and EVs, the serum (Fig. 4) and local colonic (Fig. 5) concentrations of IL-6, TNF-α, IFN-γ, IL-17, or IL-12 returned to pre-DSS baseline levels while the serum and local concentration of IL-10 significantly increased as compared to the PBS treated mice.

### MSCs and EVs physically associate with JNK1/2 and STAT3 activation

**Fig. 6:**
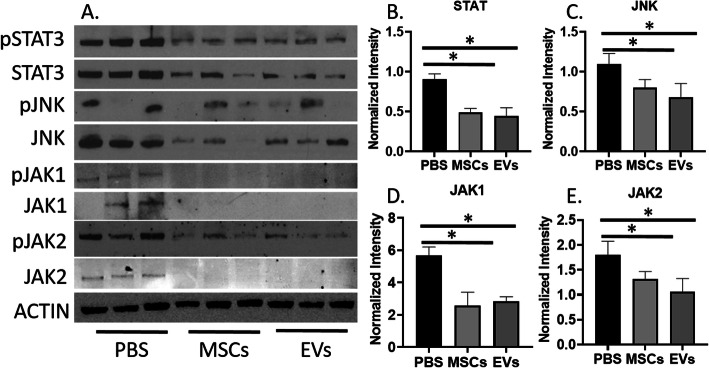
**a** MSCs/EVs inhibited the expression of STAT3, JNK, JAK1, and JAK2 after treatment. **b**–**e** The results of western blots are measured by Image J. Graphs show the results relative to β-actin. Data are shown as means ± SEM from three independent experiments, *p < 0.05

Increased evidence indicates that the levels of activated Stat3, JNK, and JAK signaling are directly correlated with the degree of intestinal inflammation in humans with IBD [43–47]. In our study, the Western blot analysis of colon protein lysates demonstrated the phosphorylated and total JNK1/2 and STAT3 protein levels were reduced in the MSC- and EV-treated groups as compared to the PBS group (Fig. 6a). The protein levels of phosphorylated JNK1/2 and STAT3 were normalized by the internal control β-actin, indicating that the observed effects on the aforementioned proteins were not caused by a nonspecific reduction of protein expression (Fig. 6b, c; *p* < 0.05).

### MSCs and EVs specifically inhibit JAK activation

The difference of JAK1 and JAK2 after MSC and EV treatment was determined with Western blot (Fig. 6a). Analysis of the phosphorylated JAK1 and JAK2 protein expression in the mice colon protein lysates demonstrated that MSC- and EV-treated mice had significantly reduced JAK1 and JAK2 expression levels as compared to the PBS controls, while normalized total expression level of JAK1 and JAK2 remained stable (Fig. 6d, e). MSCs and EVs had no significant difference on regulating the JAK1 and JAK2 expression level.

## Discussion

While MSCs have become an increasingly utilized novel therapeutic for the treatment of perianal fistulizing CD, there are several limitations of cell-based therapy including cost and scalability of manufacturing, short shelf life, and cell-to-cell variability in efficacy which can all lead to prohibitive costs and low reproducibility [48, 49]. Thus, there has been significant consideration for how to overcome these limitations of cell-based therapy. One area of increasing interest is acellular therapy, which includes the microparticles secreted by MSCs termed extracellular vesicles (EVs). Because MSCs are thought to function in a paracrine fashion, EVs, which carry mRNA, miRNA, and proteins from MSCs, are thought to carry out the paracrine effects of MSCs and therefore be the functional portion of MSCs. However, MSCs and EVs have yet to be directly compared in clinical trials, or even in specific animal models representing human disease states. To investigate therapeutics for the treatment of IBD, a colitis model can be used. When we treated a DSS-induced colitis murine model with MSCs, EVs, and placebo, we found MSCs and EVs were equivalent in their ability to reverse DSS colitis as evident by colon length, colonic bleeding, and histologic severity of inflammation.

There is increasing evidence to support MSCs repair tissue via a paracrine mechanism [23, 50]. It is thought that the paracrine mechanism of action by MSCs is carried out through the horizontal transfer of mRNA or microRNAs shuttled by EVs to target cells [16, 32, 50, 51] where they subsequently alter gene and protein expression [52]. This suggests that rates of tissue repair should be equivalent when either MSCs or EVs are delivered to an area of injury. The emerging data regarding cell-to-cell communication with extracellular vehicles (EVs) is quickly gaining traction; EVs are emerging as essential mediators for immune modulation when their molecular cargo is delivered. In the past decade, it has been proven that genetic information transporters (i.e., microRNAs) can alter genetic expression of a target cell [53]. Expanding on this has been the fact that mRNA- and microRNA-enriched EVs are able to result in recipient cell alteration [54, 55]. EVs containing miRNAs (EV-miRNAs) which can be systemically transported by blood or other body fluids present a likely role in both local and systemic intercellular information transmission [56]. This offers several advantages when thinking about the clinical application of EVs in the future. First, this is an acellular product so there would be no concern of cellular contamination with oncogenic cells or uncontrolled cell division. Second, MSCs could be cultured under a variety of conditions or engineered to produce a more immunosuppressive EV product, or a product specifically tailored to a particular disease state [57–59]. Third, there could be higher yield of product than requiring an invasive harvest of MSCs each time a cell bank is needed for a trial, and EVs are more stable to transfer. Fourth, MSCs are responsive to environmental changes, showing variable secretion profiles and phenotypes upon different in vitro stimulation [60, 61]; EVs would be a consistent product regardless of the microenvironment and could even travel systemically for distant cell signaling. In addition, when interacting with specific cells, EVs that carry cytokines have the ability to carry out multiple biologic activities [62, 63]. For these reasons, there has been an increasing interest in utilizing EVs as a clinical therapeutic. However, despite the increased number of clinical trials in CD using MSCs [13–15] [17] and the first few clinical trials using EVs [64, 65], there has yet to be a trial studying MSC-derived EVs for the treatment of IBD.

Specific to IBD, there have now been several animal studies highlighting the effectiveness of MSCs and potential pathways explaining their ability to regenerate and heal colonic tissue [18, 66, 67]. Recently, there have also been several papers reporting the success of EVs for the treatment of DSS-induced colitis in a murine model [68, 69]. Proposed mechanisms include the ability of EVs to regulate inflammatory cell polarization and apoptosis, and involve JAK1/STAT1/STAT6/Caspase signaling pathway regulation. However, there has yet to be a study directly comparing MSCs and the MSC-derived EVs to ensure equivalent efficacy. We herein found not only equivalent rates of colonic healing, but also equivalent systemic cytokine delivery and proposed mechanisms of action including suppressed inflammatory cell migration, increased antioxidant activity, and regulated apoptosis/inflammation signaling.

Because IBD is the consequence of a dysregulated mucosal immune system and uncontrolled inflammation, studying how EVs may alter these inflammatory pathways provides insight into the mechanism of MSC- and MSC-EV-induced healing. Previous publications have indicated that JNK1/2 signaling pathway-mediated regulation of STAT3 activation is linked to the development of local inflammation [70, 71]. We therefore examined the expression of JNK1/2 and STAT3 in the colon protein lysates after the delivery of MSCs, EVs, and PBS control. Consistent with our hypothesis, we found that total JNK1/2 and total STAT3 protein levels were reduced in the MSC- and EV-treated groups as compared to the PBS group, again underscoring the equivalency in MSC and MSC-derived EV treatments, highlighting this pathway as a potential target of MSC and MSC-derived EV mechanism of action.

In addition to modifying local inflammation, MSCs and EVs are thought to affect the local cellular microenvironment. The Janus kinase/signal transducers and activators of transcription (JAK/STAT) pathway is one of key pleiotropic cascades used to transduce a multitude of signals which regulates inflammation [72]. In general terms, the JAK/STAT signaling pathway is the principal signaling mechanism for analysis of the cytokine production [73–75]. Furthermore, cell proliferation, differentiation, and apoptosis are regulated by JAK signaling [76–80]. To provide further evidence that MSCs and EVs modulate JAK signaling, we analyzed the JAK1 and JAK2 expression in the mouse colon protein lysates. Compared with the PBS control group, MSC- and EV-treated mice had significantly reduced JAK1 and JAK2 expression levels, while the protein levels of β-actin remained stable. Therefore, MSCs and EVs demonstrated an equivalent function to inhibit JAK signaling activation and thus equal ability to suppress local inflammation which highlights that EVs may provide a novel acellular therapeutic with equivalent function to that of MSCs.

While this is the first investigation to directly compare MSCs and MSC-derived EVs in a murine model of colitis, there are several limitations to our paper that are worth mentioning. The first is we only used healthy donors’ MSCs and EVs from adipose-derived tissue for therapeutic purposes. In the future, it may be worth evaluating bone marrow- or umbilical-derived MSCs and EVs and MSCs from various donors to ensure the data is consistently replicated. Second, tofacitinib, a JAK inhibitor, was recently approved for the treatment of moderate to severe UC and is being increasingly used in clinical practice. Thus, it may be worth understanding if there is a synergistic effect when administered with MSCs or EVs given the reduced JAK1/JAK2 expression levels seen. Third, various doses of MSCs and concentrations of EVs were not compared; there may be a treatment-dependent dose worth exploring in the future. Despite these limitations, there is clearly increasing interest in utilizing EVs for clinical trials, seen by the increasing number clinical trials being added to ClinicalTrials.gov such as the safety of intracoronary infusion of EVs for patients with acute myocardial infarction (NCT04327635) and the study of intravenous delivered stem cell-derived EVs in preterm neonates at high risk for bronchopulmonary dysplasia (NCT03857841).

## Conclusion

In summary, we have found that MSCs and EVs have equivalent efficacy in reversing murine DSS-induced colitis, suggesting they may have equivalent therapeutic efficacy in future human clinical trials of inflammatory bowel disease.

## Supplementary Information

**Additional file 1: Figure S1.** Zetaview Nanoparticle Tracking Analyzer captured EVs. Compared to the EV group, the analyzer did not identify visible EVs in the PBS and media control group. Upper panel: the pictures show the visible particles were detected in the EV group. The other groups (PBS and Media control) cannot be detected in sample pools due to the decreased sensitivity. Lower panel: The size of the detected particles is shown.

## Data Availability

The data that support the findings of this study are included in this manuscript, and the original files are available from the corresponding author upon reasonable request.

## Abbreviations

IBD: Inflammatory bowel disease
UC: Ulcerative colitis
CD: Crohn’s disease
MSCs: Mesenchymal stem cells
EVs: Extracellular vesicles
DSS: Dextran sulfate sodium
O.C.T.: Optimal cutting temperature
H&E: Hematoxylin and eosin
TMB: Tetramethylbenzidine
SOD: Superoxide dismutase
