# Supplementary figures and images for: Mesenchymal stem cells and acellular products attenuate murine induced colitis
Source: Stem Cell Res Ther. 2020 Nov 30;11:515. doi: 10.1186/s13287-020-02025-7 (PMC7706051; doi:10.1186/s13287-020-02025-7)

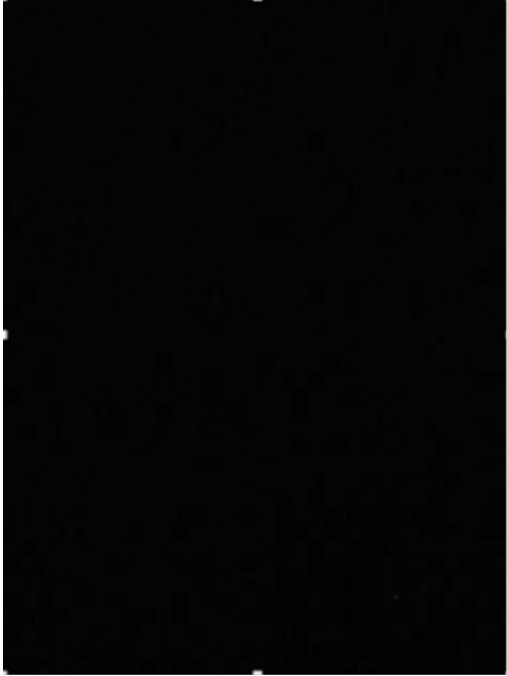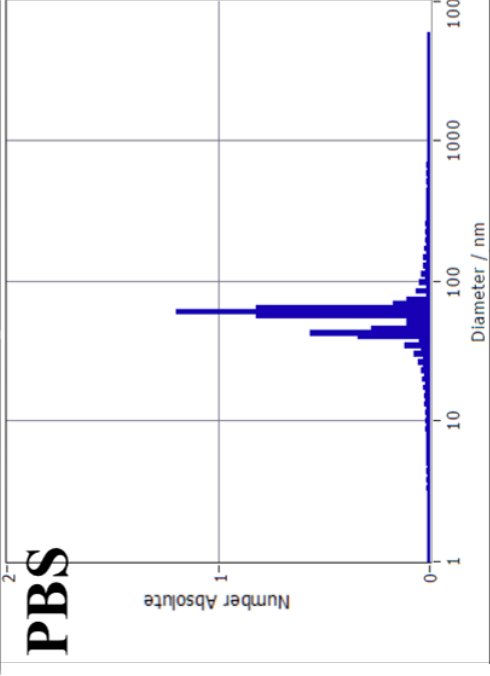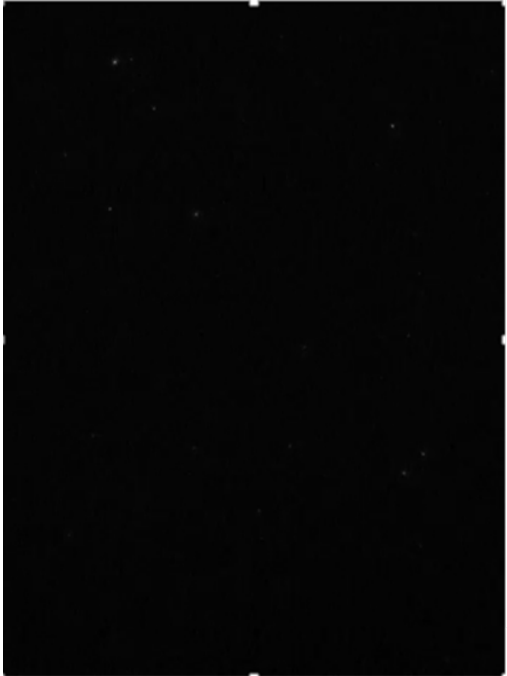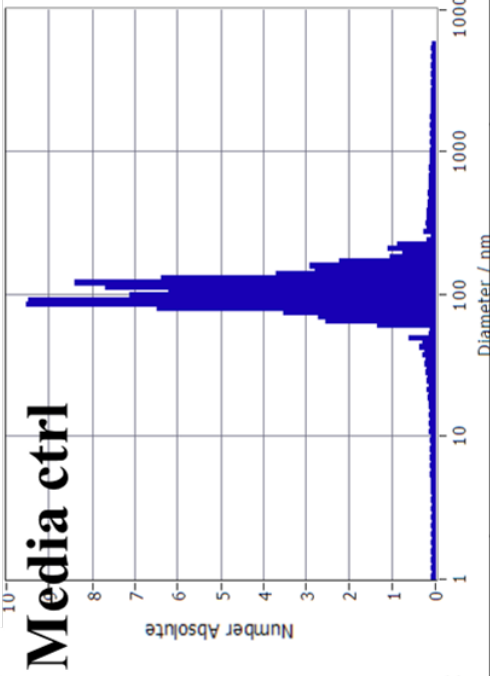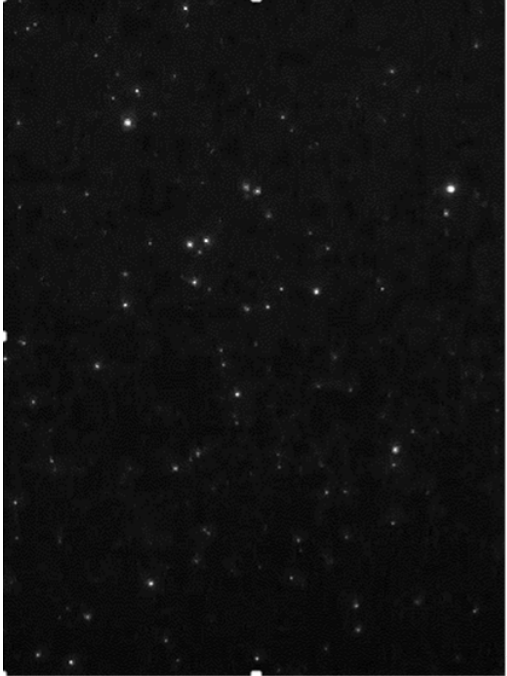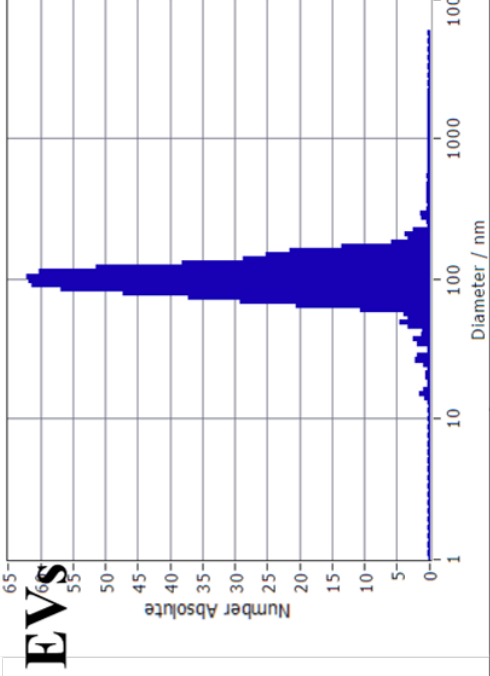

|            | Diameter/nm | Number Absolute | FWHM/nm | Percentage |
|------------|-------------|-----------------|---------|------------|
| PBS        | 43.3        | 0.5             | 5.1     | 89.9       |
| Media ctrl | 90.7        | 9.5             | 19.7    | 90.1       |
| EVs        | 100         | 61.9            | 65.8    | 100        |

Supplement: Supplementary file 1 — Additional file 1: Figure S1. Zetaview Nanoparticle Tracking Analyzer captured EVs. Compared to the EV group, the analyzer did not identify visible EVs in the PBS and media control group. Upper panel: the pictures show the visible particles were detected in the EV group. The other groups (PBS and Media control) cannot be detected in sample pools due to the decreased sensitivity. Lower panel: The size of the detected particles is shown. [file 13287_2020_2025_MOESM1_ESM.pdf]
